# Orbital IgG4-Related Disease: Clinical Features and Diagnosis

**DOI:** 10.5402/2012/412896

**Published:** 2012-06-21

**Authors:** Toshinobu Kubota, Suzuko Moritani

**Affiliations:** ^1^Department of Ophthalmology, National Hospital Organization Nagoya Medical Center, 4-1-1, Sannomaru, Naka-ku, Nagoya-shi, Aichi-ken 460-0001, Japan; ^2^Department of Pathology, National Hospital Organization Nagoya Medical Center, 4-1-1, Sannomaru, Naka-ku, Nagoya-shi, Aichi-ken 460-0001, Japan

## Abstract

Orbital IgG4-related disease, which can occur in adults of any age, is characterized by IgG4-positive lymphoplasmacytic infiltrations in ocular adnexal tissues. The signs and symptoms include chronic noninflammatory lid swelling and proptosis. Patients often have a history of allergic disease and elevated serum levels of IgG4 and IgE as well as hypergammaglobulinemia. Orbital IgG4-related disease must be differentiated from idiopathic orbital inflammation and ocular adnexal marginal zone B-cell lymphoma to ensure appropriate and effective treatment. Systemic steroid therapy decreases the size of the lesions, but relapse often occurs when systemic steroid therapy is discontinued.

## 1. Introduction

IgG4-related diseases are systemic syndromes characterized by elevated serum levels of IgG4 and IgG4-positive lymphoplasmacytic infiltrative lesions in the body. Orbital tissues are affected by IgG4-related conditions. It was first observed that Mikulicz's disease correlated with IgG4-related disease [1] and later determined that IgG4-related disease can occur in any ocular adnexal tissues [2–5]. Here, we review the clinicopathological features, differential diagnosis, and treatments of orbital IgG4-related disease on the basis of a meta-analysis of 42 patients including 3 case series studies.

## 2. Clinical Presentation

The median age of patients with orbital IgG4-related disease is 59 years (range: 30 to 86 years) with a 1 : 1 male-to-female ratio [3–5]. Notably, there is a 1 : 3 for bilateral lacrimal lesions similar to finding in Mikulicz's disease [1]. Although orbital IgG4-related disease can occur in men and women of any age, many patients have a history of allergic diseases such as asthma and allergic rhinitis. 

**Figure 1:**
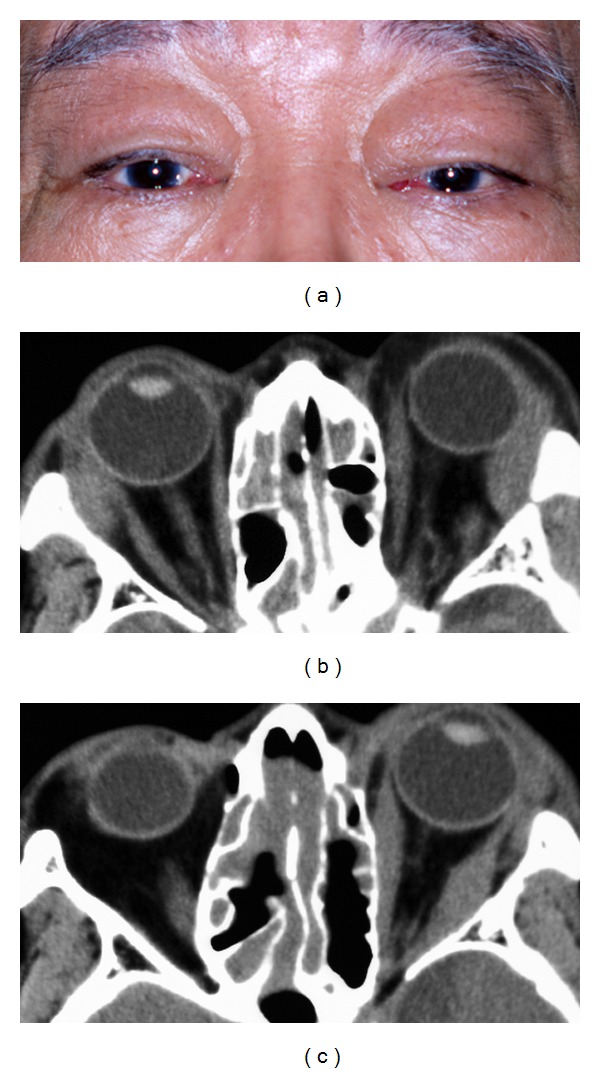
A typical case of orbital IgG4-related disease. A 72-year-old man with bilateral upper eyelid swellings that developed over a one-year period (a). There was a 10-year history of chronic rhinosinusitis, and the patients had undergone surgical treatments. On examination, his best-corrected visual acuity was 20/20 OD and 20/25 OS. Computed tomography showed enlargements of both lacrimal glands (b), enlargement of several of the left extraocular muscles (c), and infiltrative lesions in the ethmoid and maxillary sinuses. However, his ocular movements were unrestricted, and diplopia was not observed. He had elevated levels of serum IgG (4205 mg/dL; normal range: 870–1700 mg/dL) and serum IgG4 (1190 mg/dL; normal range: 4.8–105 mg/dL). These infiltrative lesions decreased in size after administration of oral prednisolone (30 mg) with a slow taper.

**Figure 2:**
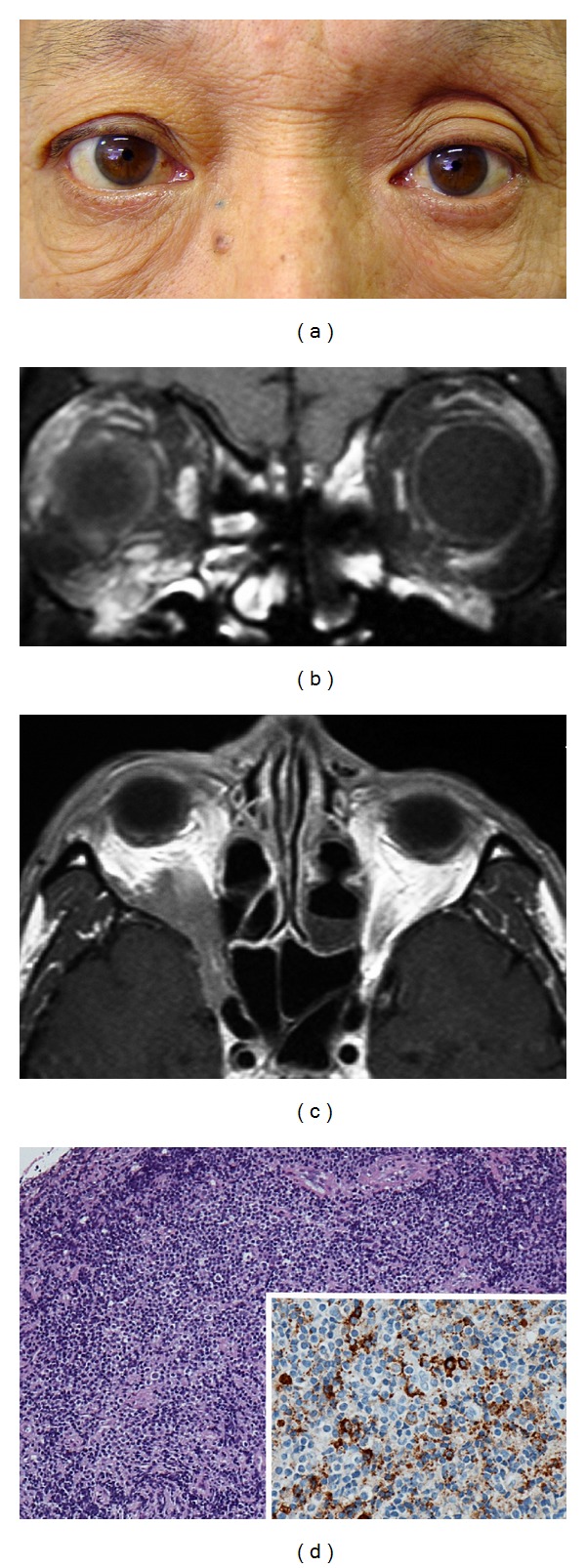
An extraorbital extension of orbital IgG4-related disease. This 60-year-old man presented with visual field defects in the right eye during a one-month period. On examination, his best-corrected visual acuity was 20/20 OD and 20/12.5 OS. Humphrey 30-2 threshold perimetry on his right eye showed an inferior altitudinal defect. He had 4 mm proptosis in the right eye (a). Magnetic resonance imaging of the brain and orbits with gadolinium showed infiltrative lesions in the right inferior orbit, infraorbital nerves (b), and also in the orbital apex and cavernous sinus (c). Clinical findings and imaging studies suggested compressive optic neuropathy. He had an elevated level of serum IgG4 (223 mg/dL; normal range: 4.8–105 mg/dL). Biopsy specimens showed lymphoproliferative lesions with IgG4-positive plasma cells and focal sclerosis (d). These findings were consistent with orbital IgG4-related disease with an extraorbital extension.

The signs and symptoms of orbital IgG4-related disease are chronic lid swelling (Figure 1) and proptosis (Figure 2), but otherwise there are only mild signs, or no signs of inflammation or periocular pain. Ocular motility is restricted mildly if at all, despite the presence of one or more enlargements of the large extraocular muscles (Figure 1). There are generally no visual disturbances, although they may occur due to apical orbital lesions (Figure 2). Imaging studies show infiltrative lesions in ocular adnexal tissues such as the lacrimal glands (Figure 1) [2–5], extraocular muscles (Figure 1) [3, 4], infraorbital nerves (Figure 2) [4], optic nerve sheath [4], lacrimal sac [6], and even cavernous sinus (Figure 2) or the intracranial extension [4]. In cases of orbital IgG4-related disease, 62% have bilateral lesions, 69% have lacrimal gland involvement, and 48% have bilateral lacrimal gland involvement [3–5].

**Figure 3:**
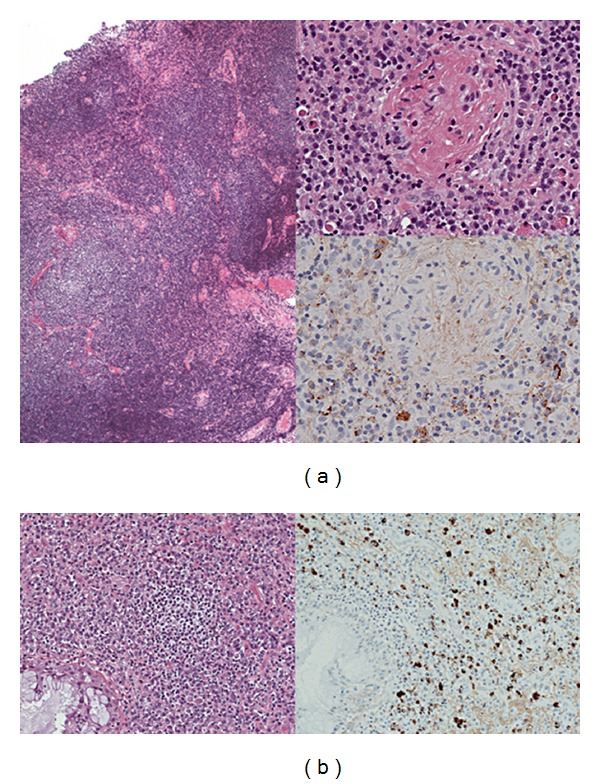
Histological findings in a typical case of orbital IgG4-related disease. Biopsy specimens from the patient in Figure 1 showed reactive lymphoid hyperplasia with plasma cells in the left lacrimal gland (a) and intensive lymphoplasmacytic infiltrations with IgG4-positive plasma cells in the ethmoid sinus (b). Immunostaining for IgG4 showed IgG4-positive plasma cells in the left lacrimal gland (top insert) and also in the ethmoid sinus (bottom, right).

Patients with orbital IgG4-related disease sometimes have systemic IgG4-related lesions in their submandibular glands (29%), lymph nodes (14%), pancreas (5%), or bile ducts (5%) [3–5]. IgG4-related lesions in the thyroid and pituitary may also be present [7, 8]. Chronic rhinosinusitis with orbital IgG4-related lesions has similar histology (Figure 3), although nonspecific chronic rhinosinusitis can be also associated with IgG4-positive plasma cells in the lesions [9].

Laboratory data of patients with orbital IgG4-related disease show elevated serum levels of IgG4 and IgE, as well as hypergammaglobulinemia [2–5]. In contrast, some patients have extremely low serum levels of IgE [3]. High IgG4 levels are associated with elevated levels of the soluble interleukin (IL-2) receptor [3]. Sclerosing pancreatitis and cholangitis, considered as IgG4-related diseases, have lymphocytes that signal for IL-4 and IL-10 *in situ* [10]. Although an etiology for the IgG4-related group of disease has been not determined yet, one plausible hypothesis for these serological and immunological abnormalities is that they result from *in vivo* activation of the immune system by activated Th2 cells [3, 10].

## 3. Histology 

The histology of orbital IgG4-related disease includes different degrees of lymphoplasmacytic infiltration with dominant sclerosing lesions or reactive lymphoid follicle (reactive lymphoid hyperplasia, Figure 3) [2–5]. Eosinophilic infiltrations are also observed [3, 4]. Rarely, orbital IgG4-related disease may have an infiltration by lymphoplasmacytic cells and macrophages containing eosinophilic material [3]. IgG4-related diseases in the body are characterized histologically by obliterative phlebitis; however, it is rare in orbital IgG4-related disease [11]. Immunohistochemical analysis shows IgG4-positive plasma cells (Figure 3), which differentiate IgG4-related disease from other inflammatory conditions arising from the ocular adnexa [3, 4].

## 4. Differential Diagnosis

**Figure 4:**
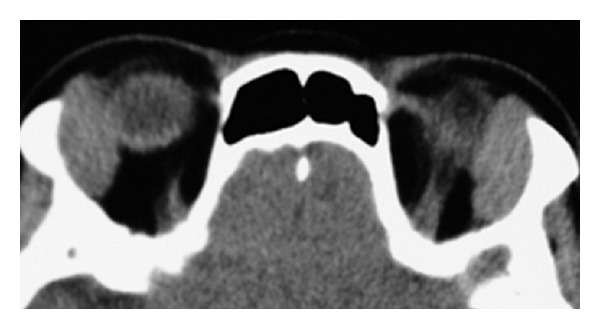
An unrelated case with symptoms similar to orbital IgG4-related disease. This 71-year-old woman had a 6-month history of swelling of both upper eyelids. Computed tomography showed an enlargement of both lacrimal glands. Histology and immunohistochemistry showed reactive lymphoid hyperplasia without IgG4-positive plasma cells. The woman had normal level of IgG4 (57 mg/dL) and an indolent clinical course for two years without treatments.

For optimal treatment and resolution, orbital IgG4-related disease must be differentiated from the following: idiopathic orbital inflammation, idiopathic orbital myositis, marginal zone B-cell lymphoma, antineutrophil cytoplasmic antibody- (ANCA-) mediated systemic vasculitis (such as Churg-Strauss syndrome and Wegener granulomatosis), and reactive lymphoid hyperplasia without IgG4-positive plasma cells (Figure 4).

Idiopathic orbital inflammations and idiopathic orbital myositis have unknown etiology but involve inflammation. They are characterized by sudden onset of orbital inflammation, periocular pain, swelling and redness of the eyelids, proptosis, ptosis, and ocular motility restrictions [12]. These differ from the signs and symptoms of orbital IgG4-related disease. However, some cases of idiopathic orbital inflammation have atypical signs and symptoms, that is, they have lacked acute onset and inflammatory signs. In such cases, biopsy specimens are needed to differentiate idiopathic orbital inflammation from IgG4-related disease. Idiopathic orbital inflammations show lymphoplasmacytic infiltration and fibrosis with few IgG4-positive plasma cells [3].

 Ocular adnexal marginal zone B-cell lymphomas make up the majority of lymphomas arising from the ocular adnexa. They are characterized histologically by the presence of reactive follicles in up to 64% of cases, sclerosis in up to 20% of cases, and plasma cells in up to 35% of cases [13]. These histological characteristics are similar to those of orbital IgG4-related disease. In addition, 9% of patients with ocular adnexal marginal zone B-cell lymphomas have infiltration of IgG4-positive plasma cells and elevated serum level of IgG4 [14]. Evidence of light chain restrictions by *in situ* hybridization and immunoglobulin heavy chain gene rearrangements by southern blot analysis can differentiate between marginal zone B-cell lymphomas with IgG4-positive plasma cells and IgG4-related inflammatory disorder. Ocular adnexal lymphomas are reported to arise in IgG4-related sclerosing dacryoadenitis, indicating a possible link between the two conditions [15]. However, the causal relationship between lymphomas and IgG4-related disease remains unclear. 

 ANCA-related vasculitis often infiltrates in ocular adnexal lesions. The symptoms of patients with orbital lesions include periocular pain, which can differentiate these patients from those with orbital IgG4-related disease. However, the histology may be similar to that of IgG4-related disease. Thus, ANCA-related vasculitis may not only include nonspecific inflammatory lesions [16], but also have abundant IgG4-positive plasma cells [17].

Finally, Mikulicz's disease includes symmetrical bilateral lacrimal gland enlargements and frequently correlates with IgG4-related disease [1]. However, symmetrical bilateral lacrimal gland enlargements do not always indicate IgG4-related lesions (Figure 4).

## 5. Treatments

Treatments for patients with orbital IgG4-related diseases may include systemic steroids, radiotherapy, or rituximab [3–5]. Studies of each of these treatments involved relatively few patients, making it difficult to evaluate the treatment outcomes via meta-analysis. Orbital IgG4-related diseases resolve after systemic steroid therapy, but relapse is often observed following therapy discontinuation. In Mikulicz's disease, relapses of lesions are observed when steroids were discontinued. Therefore, it may be best to continue prednisolone at 5 to 10 mg/day or to combine prednisolone with an immunosuppressant such as azathioprine [18]. Rituximab treatment leads to prompt clinical and serologic improvement in patients with refractory IgG4-related diseases [19], although recurrence is also observed after rituximab treatment ends in some patients with orbital IgG4-related disease [4].

## 6. Conclusions

Orbital IgG4-related disease has several unique characteristics that distinguish it from other orbital inflammatory conditions. Orbital IgG4-related disease differs from other IgG4-related diseases in the body in that it arises from nonglandular lesions and is not associated histologically with obliterative phlebitis.

## References

[1] Yamamoto M, Takahashi H, Sugai S, Imai K (2005). Clinical and pathological characteristics of Mikulicz’s disease. *Autoimmunity Reviews*.

[2] Takahira M, Kawano M, Zen Y, Minato H, Yamada K, Sugiyama K (2007). IgG4-related chronic sclerosing dacryoadenitis. *Archives of Ophthalmology*.

[3] Kubota T, Moritani S, Katayama M, Terasaki H (2010). Ocular adnexal IgG4-related lymphoplasmacytic infiltrative disorder. *Archives of Ophthalmology*.

[4] Plaza JA, Garrity JA, Dogan A, Ananthamurthy A, Witzig TE, Salomão DR (2011). Orbital inflammation with IgG4-positive plasma cells: manifestation of IgG4 systemic disease. *Archives of Ophthalmology*.

[5] Sato Y, Ohshima KI, Ichimura K (2008). Ocular adnexal IgG4-related disease has uniform clinicopathology. *Pathology International*.

[6] Batra R, Mudhar HS, Sandramouli S (2012). A unique case of IgG4 sclerosing dacryocystitis. *Ophthalmic Plastic Reconstractive Surgery*.

[7] Jakobiec FA, Stacy RC, Hatton MP (2010). Clinical characterization and immunopathologic features of sclerosing dacryoadenitis and Riedel thyroiditis. *Archives of Ophthalmology*.

[8] Patel SM, Szostek JH (2011). IgG4-related systemic disease in a native American man. *Internal Medicine*.

[9] Moteki H, Yasuo M, Hamano H, Uehara T, Usami SI (2011). IgG4-related chronic rhinosinusitis: a new clinical entity of nasal disease. *Acta Oto-Laryngologica*.

[10] Zen Y, Fujii T, Harada K (2007). Th2 and regulatory immune reactions are increased in immunoglobin G4-related sclerosing pancreatitis and cholangitis. *Hepatology*.

[11] Sato Y, Notohara K, Kojima M, Takata K, Masaki Y, Yoshino T (2010). IgG4-related disease: historical overview and pathology of hematological disorders: review Article. *Pathology International*.

[12] Kubota T, Gran JT (2011). Orbital myositised. *Idiopathic Inflammatory Myopathies-Recent Developments*.

[13] Ferry JA, Fung CY, Zukerberg L (2007). Lymphoma of the ocular adnexa: a study of 353 cases. *American Journal of Surgical Pathology*.

[14] Kubota T, Moritani S, Yoshino T, Nagai H, Terasaki H (2010). Ocular adnexal marginal zone B cell lymphoma infiltrated by IgG4-positive plasma cells. *Journal of Clinical Pathology*.

[15] Cheuk W, Yuen HKL, Chan ACL (2008). Ocular adnexal lymphoma associated with IgG4+ chronic sclerosing dacryoadenitis: a previously undescribed complication of IgG4-related sclerosing disease. *American Journal of Surgical Pathology*.

[16] Bijlsma WR, Hené RJ, Mourits MP, Kalmann R (2011). Orbital mass as manifestation of Wegener’s granulomatosis: an ophthalmologic diagnostic approach. *Clinical and Experimental Rheumatology*.

[17] Yamamoto M, Takahashi H, Suzuki C (2010). Analysis of serum IgG subclasses in churg-strauss syndrome-The meaning of elevated serum levels of IgG4. *Internal Medicine*.

[18] Yamamoto M, Takahashi H, Ohara M (2006). A new conceptualization for Mikulicz’s disease as an IgG4-related plasmacytic disease. *Modern Rheumatology*.

[19] Khosroshahi A, Bloch DB, Deshpande V, Stone JH (2010). Rituximab therapy leads to rapid decline of serum IgG4 levels and prompt clinical improvement in IgG4-related systemic disease. *Arthritis and Rheumatism*.

